# Hemosiderin Accumulation in Liver Decreases Iron Availability in Tachycardia-Induced Porcine Congestive Heart Failure Model

**DOI:** 10.3390/ijms23031026

**Published:** 2022-01-18

**Authors:** Monika Kasztura, Liliana Kiczak, Urszula Pasławska, Jacek Bania, Adrian Janiszewski, Alicja Tomaszek, Maciej Zacharski, Agnieszka Noszczyk-Nowak, Robert Pasławski, Aleksandra Tabiś, Piotr Kuropka, Piotr Dzięgiel, Piotr Ponikowski

**Affiliations:** 1 Research and Development Centre, Regional Specialist Hospital in Wroclaw, 51-124 Wroclaw, Poland; monika.kasztura@upwr.edu.pl (M.K.); urszula.paslawska@umk.pl (U.P.); jacek.bania@upwr.edu.pl (J.B.); adrian.janiszewski@up.poznan.pl (A.J.); alicja.tomaszek@upwr.edu.pl (A.T.); maciej.zacharski@upwr.edu.pl (M.Z.); agnieszka.noszczyk-nowak@upwr.edu.pl (A.N.-N.); robert.paslawski@umk.pl (R.P.); piotr.dziegiel@umed.wroc.pl (P.D.); piotr.ponikowski@umed.wroc.pl (P.P.); 2 Department of Food Hygiene and Consumer Health Protection, Faculty of Veterinary Medicine, Wroclaw University of Environmental and Life Sciences, 50-375 Wroclaw, Poland; aleksandra.tabis@upwr.edu.pl; 3 Department of Biochemistry and Molecular Biology, Faculty of Veterinary Medicine, Wroclaw University of Environmental and Life Sciences, 50-375 Wroclaw, Poland; 4 Department of Internal Diseases and Clinic of Diseases of Horses, Dogs and Cats, Faculty of Veterinary Medicine, Wroclaw University of Environmental and Life Sciences, 50-375 Wroclaw, Poland; 5 Institute of Veterinary Medicine, Faculty of Biological and Veterinary Sciences, Nicolaus Copernicus University in Torun, 87-100 Torun, Poland; 6 Department of Internal Disease and Veterinary Diagnosis, Faculty of Veterinary Medicine and Animal Sciences, Poznan University of Life Sciences, 60-637 Poznan, Poland; 7 Department of Pathology, Faculty of Veterinary Medicine, Wroclaw University of Environmental and Life Sciences, 50-375 Wroclaw, Poland; 8 Department of Biostructure and Animal Physiology, Faculty of Veterinary Medicine, Wroclaw University of Environmental and Life Sciences, 50-375 Wroclaw, Poland; piotr.kuropka@upwr.edu.pl; 9 Department of Human Morphology and Embryology, Faculty of Medicine, Wroclaw Medical University, 50-367 Wroclaw, Poland; 10 Department of Heart Diseases, Wroclaw Medical University, 50-367 Wroclaw, Poland

**Keywords:** animal model, iron deficiency, assembled ferritin, ferritin-bound Fe^3+^, hepcidin

## Abstract

Despite advances in the management of iron deficiency in heart failure (HF), the mechanisms underlying the effects of treatment remain to be established. Iron distribution and metabolism in HF pathogenesis need to be clarified. We used a porcine tachycardia-induced cardiomyopathy model to find out how HF development influences hepatic and myocardial iron storing, focusing on ferritin, the main iron storage protein. We found that cumulative liver congestion (due to the decrease of heart function) overwhelms its capacity to recycle iron from erythrocytes. As a consequence, iron is trapped in the liver as poorly mobilized hemosiderin. What is more, the ferritin-bound Fe^3+^ (reflecting bioavailable iron stores), and assembled ferritin (reflecting ability to store iron) are decreased in HF progression in the liver. We demonstrate that while HF pigs show iron deficiency indices, erythropoiesis is enhanced. Renin–angiotensin–aldosterone system activation and hepatic hepcidin suppression might indicate stress erythropoiesisinduced in HF. Furthermore, assembled ferritin increases but ferritin-bound Fe^3+^ is reduced in myocardium, indicating that a failing heart increases the iron storage reserve but iron deficiency leads to a drop in myocardial iron stores. Together, HF in pigs leads to down-regulated iron bioavailability and reduced hepatic iron storage making iron unavailable for systemic/cardiac needs.

## 1. Introduction

Iron deficiency (ID) constitutes a frequent comorbidity in heart failure (HF) [1] and can occur with and without anemia [2]. As many as 40–70% of patients with congestive HF (CHF) are iron deficient [3]. The clinical benefit of treating ID in patients with CHF was reflected in the 2016 European Society of Cardiology guidelines for HF, providing recommendations to ID diagnosis and treatment [2]. Recent findings showed that efficient management of ID in HF is associated with lower risk of hospitalizations, with no apparent effect on the risk of cardiovascular death [4]. Despite remarkable advances in management of ID in HF patients based on results of clinical studies, the mechanisms underlying the observed effects of treatment remain largely unknown. Whereas the effects of systemic ID on cardiac function have been analyzed in a few rodent models [5], to our knowledge, there are no available data from large animal models showing the influence of HF development on iron metabolism. As pigs are closely related to humans in terms of remarkable heart anatomical similarity (as well as metabolic and physiological profile closeness), they seem to be suitable for translational research in the cardiovascular area [6,7]. We have established a porcine model of symptomatic chronic HF based on relatively slow, long-term right ventricular (RV) pacing [8]. This approach allows examination of myocardial and other peripheral tissue samples, parallel with relevant biometric, echocardiography, histopathological, and biochemical data.

Chronic HF is a systemic clinical syndrome with a variety of potential effects on other organ systems, i.a., a cardiohepatic syndrome [9]. The liver is very sensitive to hemodynamic changes because of its complex vascular system and high metabolic activity [10]. The effect of cardiac failure on the liver has been investigated for many decades. A number of studies have described the microscopic changes in hepatic congestion in autopsied livers and percutaneous liver biopsy specimens [11,12]. Unique to chronic HF is congestive hepatopathy featuring centrilobular congestion and sinusoidal dilatation, atrophy of centrilobular hepatocyte plates, and variable degrees of perivenular, perisinusoidal, and bridging fibrosis [12]. On the other side, the liver is a major organ of iron storage [13]. Approximately 80% of iron in hepatocytes is bound by ferritin, and 2–3% is present as heme. The remaining iron is either bound to transferrin or is present in a labile intracellular pool (LIP) [14]. Given the liver’s central role in iron homeostasis [13], there is an urgent need to clarify liver involvement in ID in HF.

As accumulating evidences indicate organ crosstalk and interaction between the heart and the liver [15], we aimed to determine the hepatic and cardiac ability to store iron along with HF development and their potential associations. In the current study, we focused on ferritin, the main iron storage and detoxification protein. Ferritin consists of heavy (FTH) and light (FTL) chain subunits that assemble into a 24-subunit hollow sphere in which iron in form of Fe^3+^ ion is sequestered. Iron storage in ferritin molecules starts from Fe^2+^ oxidation by FTH, whereas Fe^3+^ migration and the iron core formation are assisted by FTL [16]. Additionally, we analyzed hepatic expression of hepcidin, a peptide hormone, secreted primarily by hepatocytes, being a master regulator of iron homeostasis, regulating iron availability, including its recycling by macrophages, and releasing from hepatic stores [17,18]. Our aims were: (i) immunohistochemical assessment of ferritin (FTL and FTH), as well as Fe^2+^ and Fe^3+^ in hepatic and myocardial sections, (ii) assessment of the relative amount of ferritin (FTL, FTH), and Fe^3+^ in iron-loaded assembled ferritin in hepatic and myocardial homogenates, (iii) assessment of hepatic expression of hepcidin. Our experimental scheme was designed to gain an insight into the mechanism of ID during HF development.

## 2. Results

### 2.1. RV Pacing Results in a Deterioration of LV Function, Neurohormonal Activation, LV Dilatation, Heart Hypertrophy and Hepatomegaly

The pathophysiology of iron storing in HF was assessed in a porcine tachycardia-induced cardiomyopathy (TIC) model [7,17]. All the pigs included in the TIC protocol finished the study, 26 pigs assigned for the RV pacing developed HF, and the 6 control animals remained healthy. All the animals were maintained under identical environmental and dietetic conditions.

In the course of RV pacing, the pigs developed clinical signs of chronic HF and were euthanized at predefined HF stages [17], i.e., mild (6 ± 2 weeks of pacing, *n* = 9), moderate (11 ± 2 weeks of pacing, *n* = 9) and severe (20 ± 9 weeks of pacing, *n* = 8). Postmortem examination revealed cardiomegaly as well as hepatomegaly in HF pigs (Supplementary Table S1). HF symptoms were not reported in the control animals (*n* = 6). Echocardiography carried out shortly before euthanasia revealed that RV pacing induced progressive impairment of LV systolic function together with LV dilatation. Echocardiographic parameters measured in control pigs remained in the physiological range [18] (Supplementary Table S1). Furthermore, the development of symptomatic systolic HF was followed by the neurohormonal activation: increased plasma renin activity (PRA), and the higher serum levels of aldosterone, adrenaline, cortisol, and B-type natriuretic peptide (BNP) (Supplementary Table S1).

### 2.2. Paced Pigs Reveal Indices of Enhanced Erythropoiesis and ID, but with Red Blood Cells Remaining Normocytic, and Normochromic

We first examined hematological parameters and iron status indices in blood samples to get insight into possible systemic symptoms of ID. To follow the dynamic of individual red blood cell (RBC) count, hemoglobin (Hb), and hematocrit (HCT) values, we calculated the relative change of these parameters during the whole pacing period for each pig (i.e., (RBC in end-point/RBC in day 0)*100%) (Table 1). HF development was accompanied by an increase of relative RBC, Hb as well as HCT, suggesting probable enhanced erythropoiesis. Both, absolute and relative RBC, Hb and HCT correlated strongly with liver enlargement (Supplementary Table S2), AST/ALT ratio, as well as PRA and aldosterone (Supplementary Table S3). What is interesting, only relative RBC, Hb and HCT showed a positive association with LV dilatation (Supplementary Table S2). In contrast to RBC, the white blood cell count was decreasing with the development of pacing-induced HF (Table 1).

RBC remained normocytic, and normochromic during the study time (Table 1). Serum iron was decreasing along with the HF development, with approximately 60% reduction in severe HF group, in comparison to the controls (Table 1). Decreased serum iron and reduced transferrin saturation (TSAT) were accompanied by LV dilatation and systolic dysfunction, PRA and cortisol enhancement as well as liver enlargement (Supplementary Table S2). Unchanged total iron binding capacity values along with diminished TSAT and serum iron might suggest latent ID (Table 1). The development of HF was not accompanied by a raised serum ferritin (Table 1). Serum erythropoietin (EPO) measurements failed and the results remained below detection level.

### 2.3. HF Development Leads to Hepatocellular Injury with Preserved Hepatic Synthesis Capacity and Excretory Functions as Well as to Mild Systemic Oxidative Stress without Inflammation

As liver is the main organ responsible for iron storing, we performed biochemical blood parameters evaluation of its function. Serum albumin, total protein, and total bilirubin were not significantly different in HF pigs vs. controls (Table 2). Thus, the hepatic synthesis capacity and excretory functions seems to be preserved. At the same time, the ALT/AST ratio (a useful indicator of liver damage) [19] increased along with HF development, reaching values typical for hepatocellular injury in the severe HF group (Table 2) [20]. ALT/AST ratio was strongly related to liver enlargement, neurohormonal activation (PRA, aldosterone, catecholamines), and systolic dysfunction indices (Supplementary Table S3). Finally, to gain a comprehensive look, we analyzed some biochemical markers of metabolic abnormalities, inflammation and oxidative stress. HF development was accompanied by elevated glucose and triglyceride levels (Table 2). Surprisingly, there were no indices of elevated inflammatory status in HF pigs (Table 2). However, HF development was accompanied by mildly increased oxidative stress (Table 2). At the same time, the myocardial and hepatic malonyldialdehyde (MDA) level remained similar in all animals (Table 2). Hepatic aconitase activity, both in mitochondrial and cytoplasmic fraction, was decreasing with HF progression, whereas in LV, it remained unchanged (Table 2). As aconitase activity is decreased by oxidative stress or ID [21], these results suggest that hepatic ID might be responsible for diminished aconitase activity.

### 2.4. HF Development through Centrilobular Sinusoidal Congestion Leads to Accumulation of Iron-Overloaded Kupffer Cells and Centrilobular Multiple Hemosiderin Deposits, then, Hepatic Cords Atrophy with a Concomitant Drop of Kupffer Cells and Massive Hemosiderin Deposits

We evaluated the histological pattern of distribution of lesions and iron storage features focusing on the lobule, a functional unit of the liver [10] (summarized in Supplementary Tables S4 and S5).

**Figure 1:**
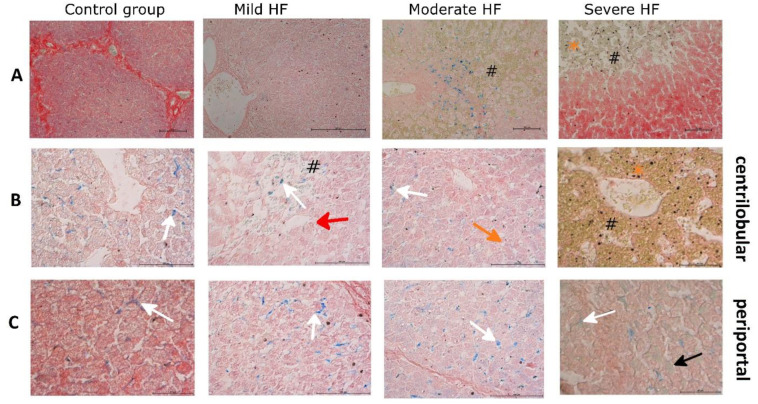
HF development leads to temporary accumulation of Fe^2+^-overloaded Kupffer cells with a final drop in severe HF. Histochemical detection of Fe^2+^ by Turnbull’s Blue reaction in liver sections from control group, pigs with mild, moderate, and severe HF. Blue staining represents Fe^2+^ accumulation in the cell, hemosiderin appears brown. (**A**) 200× original magnification, (**B**) 400× original magnification, focused on centrilobular area, (**C**) 400× original magnification, focused on the perilobular area. White arrows indicate positive reaction in macrophages, red arrows indicate dilated sinusoids in the central vein area filled with RBCs, black arrows indicate positive reaction in hepatocyte, # indicates venous congestion, orange arrow indicates dilated bile canaliculi, orange star indicates hemosiderin.

**Figure 2:**
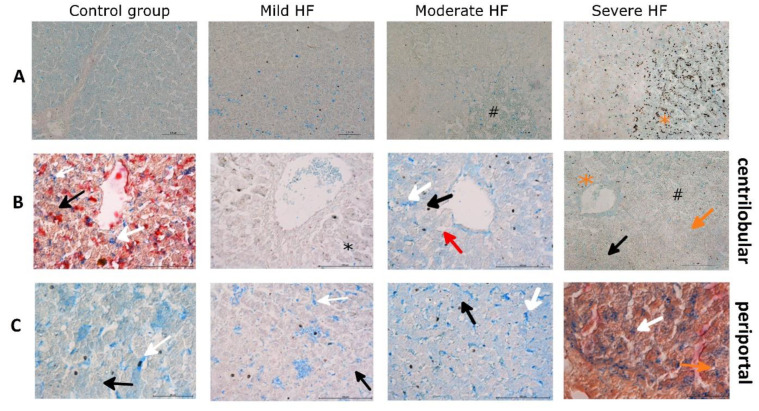
HF animals reveal increasing centrilobular sinusoidal congestion, hepatic cords atrophy, iron-overloaded Kupffer cells, up to centrilobular multiple hemosiderin (poorly mobilized form of iron) deposits with a concomitant drop of Kupffer cells in severe HF. Histochemical detection of Fe^3+^ by Prussian Blue reaction in liver sections from control group, pigs with mild, moderate, and severe HF. Iron depositions appear in blue, hemosiderin appears in brown. (**A**) 200× original magnification, (**B**) 400× original magnification, focused on the centrilobular area, (**C**) 400× original magnification, focused on the perilobular area. White arrows indicate positive reaction in macrophages, red arrows indicate dilated sinusoids in the central vein area filled with RBCs, black arrows indicate positive reaction in hepatocyte, ^#^ indicates venous congestion, orange arrows indicate dilated bile canaliculi. Black asterisk indicates Kupffer cells with hemosiderin granules; orange asterisk indicates hemosiderin.

**Figure 3:**
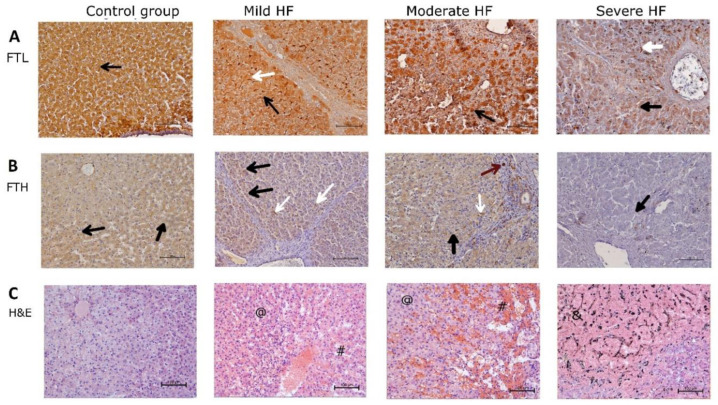
Centrilobular area in HF development is completely losing its ability to store iron, which is present in poorly available hemosiderin form. Immunohistochemical detection of ferritin and H&E staining in liver sections. (**A**) Ferritin light chain staining (FTL), (**B**) ferritin heavy chain staining (FTH), (**C**) H&E staining in control group, and pigs with mild, moderate, and severe HF. Black arrows indicate positive cytoplasmic reaction, white arrows indicate positive-stained Kupffer cells, red arrows indicate positive reaction in the lymphatic vessel wall, # indicates venous congestion, @ indicates dilated sinusoids, & indicates hemosiderin. Magnification: 200××.

HF animals revealed increasing centrilobular sinusoidal congestion in moderate HF regions representing accumulation of erythrocytes surrounded by iron-overloaded Kupffer cells (liver-resident macrophages) (Figure 1A and Figure 2A). In animals with severe HF, prominent centrilobular sinusoidal congestion and dilatation were accompanied by a compression and atrophy of the hepatic cords (Figure 3C). Within these extensive centrilobular hemorrhagic regions, multiple irregular hemosiderin deposits (poorly mobilized form of iron) were found, in some areas with a retained cord-like framework (Figure 1A, Figure 2A and Figure 3C). At the same time, a drop of iron-loaded Kupffer cells was observed (Figure 2B).

Fe^3+^ and ferritin (FTL and FTH) staining in hepatocytes was evenly distributed throughout the lobule in control and mild HF group (Figure 2 and Figure 3A,B), reflecting the undisturbed iron storing capability and availability. In moderate and severe HF animals, FTL staining with similar intensity was retained in periportal hepatocytes (Figure 3A) accompanied by faint Fe^3+^ staining (Figure 2C). In pigs with moderate HF, few preserved hepatocytes cords inside the centrilobular congestion areas showed very strong positive ferritin (FTL and FTH) staining as well as granular deposits (hemosiderin) (Figure 3A,B), while in severe HF, multiple irregular ferritin granular deposits within centrilobular regions were found (Figure 3A,C). These findings suggest that the centrilobular area in HF development is completely losing its ability to store iron, which is present in poorly available hemosiderin form.

Although the centrilobular architecture was distorted, there were no signs of the lymphocyte infiltration or collagen deposition (no development of pericentral or bridging fibrosis) as well as cholestasis in HF pigs (Figure 3C). Moreover, hepatic proinflammatory cytokines levels were decreasing along with the HF development (IL-1β, R = −0.35, *p* = 0.05; IL-6, R = −0.42, *p* = 0.02), confirming that the liver did not become inflamed.

### 2.5. Porcine Myocardium Reveals Ultrastructural Changes Typical for HF

We performed the histological and immunochemical staining in myocardium sections to evaluate iron storing and histopathological lesions in failing heart. In all control and HF animals, Fe^2+^ staining was negative (Supplementary Figure S1). Fe^3+^ staining was positive in about half of the animals, in all groups (Supplementary Figure S1). Faint diffuse blue staining in some cardiomyocytes seems to be not related to the HF progression.

**Figure 4:**
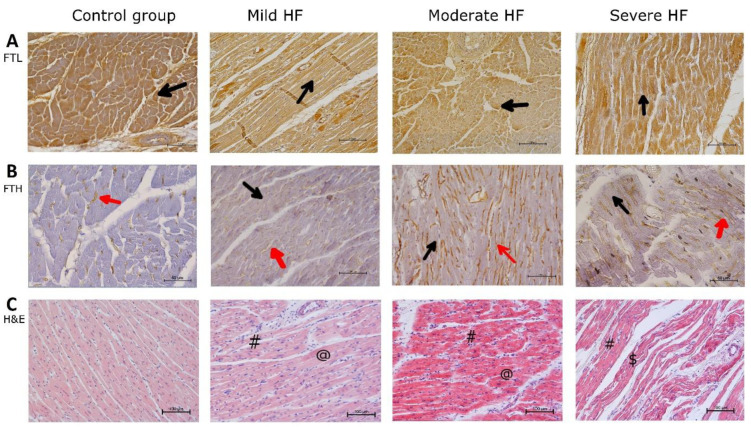
Porcine myocardium shows structural changes typical for HF but without signs of iron overload. Immunohistochemical detection of ferritin and H&E staining in LV sections. (**A**) Ferritin light chain staining (FTL), (**B**) ferritin heavy chain staining (FTH), (**C**) H&E staining in control group, and pigs with mild, moderate, and severe HF. Black arrows indicate positive reaction in cardiomyocytes, red arrows indicate positive reaction in cardiac blood capillary vessel walls, # indicates venous congestion, @ indicates hypertrophied cardiomyocyte, $ indicates wavy myocardial fibers. Magnification: 200××.

LV cardiomyocytes from the sham-operated animals showed a regular structure (Figure 4C). Mild passive congestion was observed in LV samples from animals with mild HF, as well as slight elongation of some cardiomyocytes, and atrophy of others (Figure 4C). In myocardium from pigs with moderate HF, increasing passive congestion and cardiomyocyte hypertrophy were found (Figure 4C). The structure of the heart wall was disturbed in animals from the severe HF group. There was a decrease in branching of cell bundles and anastomosing of adjacent cardiac muscle fibers, loss of cardiomyocyte cross striations, and wavy myocardial fibers (Figure 4C). These degenerative features were accompanied by cardiomyocytes hypertrophy and passive congestion in capillaries surrounding cardiac muscle fibers (Figure 4C).

Immunohistochemical analysis of normal (control group) as well as all HF animal hearts revealed FTL positive staining in the cytoplasm of cardiomyocytes. The staining of cardiomyocytes appeared patchy, with some cardiomyocytes presenting stronger signal (Figure 4A). The intensity of the staining did not differ between animal groups. Immunostaining of FTH was localized mainly to cardiac blood capillary vessel walls (endothelium) in all pigs. Cardiomyocytes in all analyzed sections showed a faint cytoplasmic FTH staining (Figure 4B). In summary, these data reveal that porcine myocardium shows structural changes typical for HF but without any signs of iron overload.

### 2.6. In the Liver the Ferritin-Bound Fe^3+^ (Iron Stores), and Assembled Ferritin (Ability to Store Iron) Are Decreasing along with HF Development with Concomitant Hepcidin Suppression

**Figure 5:**
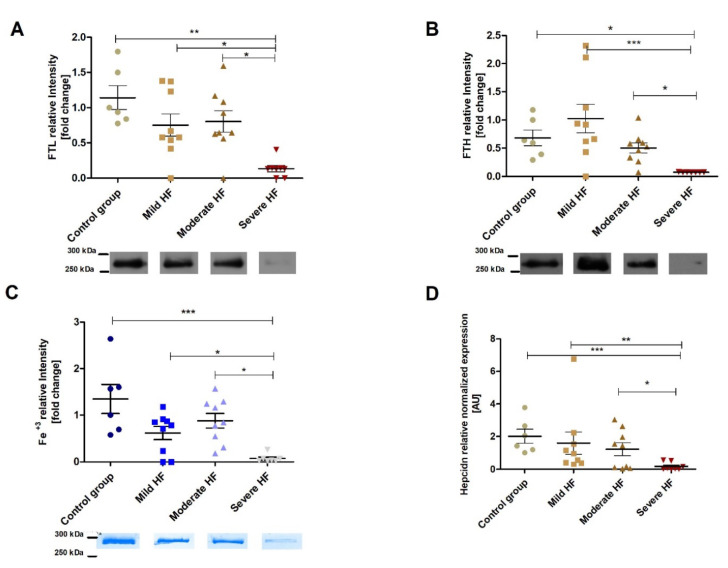
In the liver, the ferritin-bound Fe^3+^ (iron stores) and assembled ferritin (ability to store iron) decrease along with HF development with relevant hepcidin suppression. Western blot analysis of (**A**) ferritin light chain (FTL), (**B**) ferritin heavy chain (FTH), and (**C**) Prussian Blue staining detecting iron-loaded ferritin following non-reducing SDS-PAGE of hepatic ferritin-enriched supernatants. Hepatic relative levels of ferritin light (FTL), heavy (FTH) chain, and Fe^3+^ in the 300-kDa protein were analyzed in controls (*n* = 6) and animals with mild (*n* = 9), moderate (*n* = 9) and severe (*n* = 7) HF. Representative Western blots (FTH and FTL) and Fe^3+^ staining are shown below the diagrams. Results are expressed as relative band intensity and presented as the mean ± SEM. (**D**) Relative mRNA expression of hepcidin in controls (*n* = 6) and animals with mild (*n* = 9), moderate (*n* = 9) and severe (*n* = 7) HF. Differences in protein (FTL and FTH) and Fe^3+^ quantification as well as relative hepcidin expression between animal groups were assessed by the Mann–Whitney U test. * *p* < 0.05, ** *p* < 0.01 and *** *p* < 0.001.

**Figure 6:**
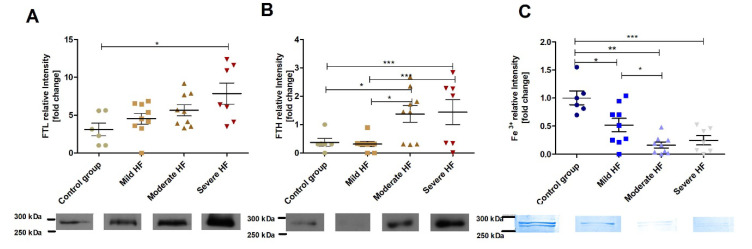
In a failing heart, the assembled ferritin (iron storage reserve) increases but ferritin-bound Fe^3+^ (iron stores) is reduced. Western blot analysis of (**A**) ferritin light chain (FTL), (**B**) ferritin heavy chain (FTH), and (**C**) Prussian Blue staining detecting iron-loaded ferritin following non-reducing SDS-PAGE of myocardial (left ventricle) ferritin-enriched supernatants. Myocardial relative levels of ferritin light (FTL), heavy (FTH) chain, and Fe^3+^ in the 300-kDa protein band were analyzed in controls (*n* = 6) and animals with mild (*n* = 9), moderate (*n* = 9) and severe (*n* = 7) HF. Representative Western blots (FTH and FTL) and Fe^3+^ staining are shown below the diagrams. Results are expressed as relative band intensity and presented as the mean ± SEM. Differences in protein (FTL and FTH) and Fe^3+^ quantification between animal groups were assessed by the Mann–Whitney U test. * *p* < 0.05, ** *p* < 0.01 and *** *p* < 0.001.

The electrophoretic analysis of hepatic and myocardial ferritin-enriched supernatants in non-reducing (using a non-reducing sample buffer) SDS-PAGE showed the presence of a prominent band corresponding to a molecular weight above 300 kDa. This band was positive for FTL, FTH, and Fe^3+^ staining (Figure 5 and Figure 6), therefore, it appears to be high-molecular-weight oligomeric iron-loaded ferritin (assembled ferritin).

As ferritin is the main iron storing protein, we analyzed the relative amount of FTL, FTH, and Fe^3+^ in this above-300-kDa protein band (assembled ferritin) found in ferritin- enriched supernatants from the liver (Figure 5) [13]. Hepatic FTL decreased along with the HF development (R = −0.59, *p* = 0.0004), being about 10 times lower in the severe HF group than in the controls (Figure 5A). Moreover, the same process was noticed for hepatic FTH (Figure 5B). As could be expected, the relative amount of ferritin-bound Fe^3+^ was also decreasing along with the progression of HF (R = −0.50, *p* = 0.004), with the difference between severe HF and control group of about 20 times (Figure 5C). Decreased hepatic ability to store iron (FTL and FTH) as well as available iron stores (ferritin-bound Fe^3+^) was related to PRA and BNP, LV dilatation, and deterioration of the heart function (Supplementary Table S3). What is more, both FTL and ferritin-bound Fe^3+^ were strongly inversely related to hepatomegaly (Supplementary Table S3). The positive strong relationship of hepatic FTL and ferritin bound-Fe^3+^ to the serum iron was also observed (Supplementary Table S3). At the same time, Ht and relative change in Hb were negatively correlated to hepatic FTL and ferritin bound Fe^3+^ (Supplementary Table S3).

As hepcidin is a key regulator of iron metabolism and is primarily produced in the liver [22], we decided to analyze its hepatic expression. A downregulation of the hepcidin expression (up to 10 times in the severe HF group as compared to the controls) (Figure 5D) was strongly associated with HF development (R = −0,62, *p* = 0.0002), and ALT/AST ratio (R = −0,62, *p* = 0.0005) as well as with hepatomegaly PRA and aldosterone (Supplementary Table S3). What is more, hepcidin expression in the liver was strongly inversely correlated with relative change of hematocrit parameters (Supplementary Table S3), and hepatic ferritin (FTL) and ferritin-bound Fe^3+^ (Supplementary Table S4). There was no association between the decline in hepatic hepcidin expression and impaired LV function and indices of iron status. In summary, hepcidin production seems to be suppressed by PRA and aldosterone and liver congestion, but not directly by an impaired heart function.

### 2.7. Increase of Myocardial Assembled Ferritin in HF Is Accompanied with Reduction of Ferritin-Bound Fe^3+^

In contrast to the liver, assembled ferritin (FTL and FTH) in LV myocardium was increasing along with HF development (Figure 6A,B). A surprising aspect of it was that, at the same time, ferritin-bound Fe^3+^ was significantly decreased (about 6 times in moderate and severe HF group vs. controls) (Figure 6C). Whereas LV ferritin (FTL and FTH) correlated positively with LV dilatation, ferritin-bound Fe^3+^ was negatively related to the same echocardiographic parameters (Supplementary Table S3). Ferritin-bound Fe^3+^ in LV was inversely related to the heart hypertrophy (Supplementary Table S3). In contrast to the liver, myocardial FTL was positively correlated to relative change in HCT and Hb (Supplementary Table S3).

## 3. Discussion

To the best of our knowledge, this is the first attempt to use a large animal HF model to determine hepatic and cardiac ability to store iron along with HF development. We have demonstrated that HF in pigs is accompanied by the occurrence of multiple irregular hepatic hemosiderin deposits within centrilobular hemorrhagic regions as well as a decreased Fe^3+^ content (observed both in the hepatocyte cytoplasm and as ferritin-bound Fe^3+^) and lower hepatic ferritin. These changes went on with the suppression of hepatic expression of hepcidin. In a failing myocardium, ferritin-bound Fe^3+^ was significantly decreased with the concomitant enhancement of assembled ferritin able to store Fe^3+^. These results suggest a downregulated biological iron availability as well as reduced hepatic iron storing ability in HF development, accompanied by an available storage reserve for iron in a failing myocardium. Assuming a high translational potential of the porcine HF model [6], proposed pathomechanism of ID in heart insufficiency might be helpful in finding new targets, i.e., the liver, in the in development of new strategies of HF treatment.

The ultrastructural changes found within the livers from severe HF animals, i.e., centrilobular congestion and sinusoidal dilatation, atrophy of centrilobular hepatocyte plates, were consistent with congestive hepatopathy indices, typical for human CHF [12]. The only difference was the lack of any signs of fibrosis. Most of the cardiovascular drugs pass through hepatic processing [19] and chronic exposure may be harmful to the liver, i.e., amiodarone often induces fibrosis and cirrhosis [20]. Thus, the lack of long-lasting pharmacological management in our HF model could explain the lack of the hepatic fibrosis in severe CHF animals. In addition, the relatively young age of our pigs could contribute to the fibrosis absence as aging increases the susceptibility of liver fibrosis [21]. At the same time, both in human chronic HF [11], and in our TIC model, the periportal zone of lobules responsible for synthetic function [22] was unaffected. Serum albumin remained unchanged as in human HF patients [6,11]. The only abnormality in the biochemical parameters of the liver function was the increased AST/ALT ratio in pigs with severe HF indicating the hepatocellular damage, consistent with the histopathological findings. In human HF, the prognostic values of liver function tests have not been established yet, as studies focusing on this issue produce conflicting results [23,24,25].

As one of our goals was to investigate hepatic ability to store iron in HF, we evaluated Fe^2+^, Fe^3+^, and ferritin subunits (FTL and FTH) in liver sections. Since we observed passive congestion in the liver which could provide a false-positive signal for iron, instead of the elemental analysis method, the iron level was assessed by histochemical methods. In the tissue, iron occurs in the form of “masked” (heme) and “not masked” Fe. From this, “masked” Fe cannot be stained by histochemistry. The “not masked” Fe includes nonheme iron complexes with proteins or low molecular weight compounds [26]. Turnbull’s blue staining is considered specific for ferrous iron (Fe^2+^), producing a pale staining in the cytoplasm of iron-rich cells such as hepatocytes or Kupffer cells [27]. Intracellular Fe^2+^ forms LIP, which can be (i) stored in ferritin, (ii) released from the cell or (iii) used in intracellular metabolism [27,28]. We observed an increase of Fe^2+^-stained Kupffer cells from mild to moderate HF, especially in the perilobular zone, and a decrease in severe HF. The level of Fe^2+^ in hepatocytes was similar in controls and HF pigs. Considering the sensitivity of Turnbull’s blue staining [26], we excluded mass Fe^2+^ accumulation in hepatocytes. To assess fine Fe^2+^ changes, we measured hepatic cytoplasmic and mitochondrial aconitase activities. These aconitase isoenzymes contain a labile iron-sulfur cluster and their activities decrease in oxidative stress or ID [29]. We revealed that the activities of both aconitases decreased along with HF development, although we did not observe an increase of hepatic MDA (oxidative stress marker). Moreover, we noted microvesicular steatosis in hepatocytes from paced pigs. According to Crooks et al., the decrease of aconitase activities accompanying ID leads to cytosolic lipid droplet formation [30]. Together, our results indicate that the HF development is associated with a decrease of LIP in hepatocytes.

It was shown that non-utilized or non-exported iron can be stored in form of Fe^3+^ in the ferritin (soluble form) or hemosiderin (insoluble form of ferritin under iron excess) [16,28]. The Fe^3+^ deposits were observed in mild/moderate HF in the Kupffer cells, then ‘spilled over’ into the hepatocytes in severe HF. Immunochemical staining revealed that both FTH and FTL occur as multiple irregular granular deposits within extensive centrilobular hemorrhagic regions in severe HF. Since immunochemical ferritin staining in periportal hepatocytes was similar in all the pigs, whereas in the centrilobular zone, ferritin was masked by hemosiderosis developing in HF, we examined assembled ferritin in liver homogenates. We showed that both FTL and FTH, as well as Fe^3+^ bound to assembled ferritin strongly decreased in HF, and negatively correlated with neurohormonal activation, and LV remodeling. Thus, HF development is accompanied by lower iron availability as well as reduced hepatic iron storing ability. Correlation of hepatic iron stores decreases, serum iron and TSAT with an increase of hematocrit and relative Hb change might indicate that iron from hepatic stores is consumed by enhanced erythropoiesis. Therefore, it seems that HF-induced hepatic passive congestion producing liver enlargement is related to a decreased iron storage ability and enhanced erythropoiesis since hepatic FTL, ferritin-bound Fe^3+^ and hematological parameters were correlated with hepatomegaly.

Efficient control of the iron level in the body likely depends on recognition, uptake, and degradation of erythrocytes by macrophages [31]. Damaged RBCs are cleared by Kupffer cells participating in heme iron recycling [32]. Fe^2+^ released from heme is either stored in assembled ferritin (after oxidation to Fe^3+^ by FTH) or exported for reuse [28]. Overwhelming of Kupffer cells’ capacity makes them die, probably by a specific form of cell death named ferroptosis [33]. Patients receiving regular RBC transfusions develop a cumulative iron overload. Initially, free iron from RBCs accumulates in Kupffer cells, then, it is deposited in the liver and heart parenchymal cells [34]. In heavily iron overloaded hepatocytes, an insoluble hemosiderin is formed from iron-rich ferritin [35]. Similar findings were noted in our porcine HF livers. However, studying the assembled ferritin level, we observed a striking difference. Whereas in the livers of patients with post-transfusional iron overload, assembled ferritin was more than fourfold more abundant than in the normal liver [36]; in our HF livers, the assembled ferritin was up to tenfold decreased in severe HF. As ferritin production is regulated by intracellular iron availability [28], it seems that in early HF, iron from congested RBC is continuously exported from the liver, thus, the hepatocytes do not get a signal to produce new ferritin molecules. In severe HF, the influx of iron from congested RBC to hepatocytes overwhelms the capacity of existing ferritin molecules to store iron and leads to hemosiderin formation in centrilobular areas.

It was shown that hematological parameters strongly correlate with HF severity and plasma renin activity (induced by renal hypoperfusion) [37]. Under hypoxic conditions, the organism upregulates the RBC production enhancing the release of EPO from kidneys, thus compensating for the low oxygen tension in the blood [28]. Volpe et al. [37] demonstrated that plasma EPO levels were increased in a large cohort of patients with CHF of various etiology, and that the EPO rise was related to disease progression. These results suggest the involvement of EPO in the complex neurohormonal response in HF. As there is a high degree of EPO homology among mammals (82% human/pig identity) [38], we used ELISA for human EPO with 50% cross-species reactivity, but the tests gave no signals. Since EPO is highly glycosylated (up to 40%) [39], the most probable reason was interspecies difference in glycosylation pattern. According to Volpe et al. [37], plasma renin activity is considered the most predictive factor of the EPO level, thus, our results could suggest that the EPO level is increased along with HF progression. In the case of chronic erythroid stress, besides EPO, an augmented cortisol level is necessary to promote a long-lasting increase in RBC production [40]. In our HF model, cortisol, as well as glucose, were elevated along with HF development. To further support our stress erythropoiesis hypothesis, we analyzed hepatic expression of hepcidin, a negative regulator of iron flows, modulated, i.a., by stress erythropoiesis [18]. Hepcidin transcription in the liver is controlled by a complex interplay of signals. It is decreased by erythroid activity to ensure iron supply for erythropoiesis, i.a., by triggering the release of iron from hepatic stores [41]. The sequence of events is as follows: EPO produced by the kidney triggers the proliferation and terminal differentiation of erythroid progenitor cells, these precursors then release an unknown soluble factor expected to communicate the increase in iron demand from the bone marrow to the liver and decrease hepcidin expression [41]. Observed suppression of hepcidin expression was strongly related to renin–angiotensin–aldosterone system (RAAS) activation and hepatomegaly, as well as hematological parameters, what is consistent with above-mentioned sequence of events. Thus, our observations strongly suggest that HF development in TIC model was accompanied by stress erythropoiesis. However, stimulation of erythropoiesis during the study time did not lead to the accumulation of hypochromic or microcytic red blood cells. According to Peyrin-Biroulet et al. [42], a TSAT level lower than 16% indicates an insufficient supply of iron for erythropoiesis, and a treatment is recommended when TSAT is <20% [43]. Animals with severe HF had TSAT of 22%, indicating they still had available iron for efficient erythropoiesis. This could explain why we did not observe any anemia indices in the severe HF group. What is more, HF was developing in our porcine model up to 4 months, whereas in human patients, this process lasts for years, and anemia is known to prevail in patients with long-term advanced HF [44].

Besides the liver, our goal was to gain an insight into the myocardial iron status. Only few studies examined directly the myocardial iron content in HF patients, suggesting iron depletion in the failing heart [44,45]. We found within porcine myocardium ultrastructural changes typical for the clinical course of human HF [46] without any signs of iron overload. Immunochemical staining for both ferritin subunits (FTH and FTL) did not show any differences between HF and control pigs. Assembled ferritin increased along with HF development with a concomitant drop of bound Fe^3+^. Decrease in myocardial iron stores did not correlate with systemic markers of iron status, similarly to the failing human myocardium [44]. These results clearly show that mechanisms of iron uptake, storage and clearance differ between liver and heart in HF progression. It also seems that the myocardial LIP remains unchanged along with HF progression, as both cytoplasmic and mitochondrial aconitase activities, as well as the MDA level in LV myocardium, were similar in the control and HF animals. Therefore, it is likely that the iron from myocardial stores might be used to meet the internal requirement of myocardium. The increase of assembled ferritin able to store Fe^3+^ could mean that a failing myocardium still has available storage reserve for iron, which can to some extent explain the beneficial effects of iron therapy in HF [43].

Our observations led us to propose a possible mechanism underlying ID in HF. HF is a condition in which the heart function is insufficient to supply tissue flow demand [47]. This causes hypoxia and stimulates erythropoiesis [28]. At the very beginning, iron, crucial for RBC production, is mobilized from hepatic stores. As the liver is very sensitive to hemodynamic changes [11], hepatic venous congestion increases (starting from the central vein). In the setting of chronic blood congestion, Kupffer cells surround the centrilobular congestion regions and start to phagocytose RBCs. The recycled iron from congested RBCs is released back into the circulation and used for production of new RBCs, supporting the iron export from hepatocytes. Increase of RBCs accompanied by the decrease of the heart function results in prolonged and cumulative hepatic congestion which overwhelms the capacity of Kupffer cells. The Kupffer cells die and atrophied hepatocytes (due to the augmented congestion) in the centrilobular zone become heavily overloaded by iron, up to the massive hemosiderin formation—they do not have enough assembled ferritin to bind the mass influx of bioavailable iron. As a consequence, hepatic iron is trapped in poorly mobilized hemosiderin form, which makes it unavailable for erythropoiesis which remains enhanced in a chronic hypoxia state. The next stage, observed in patients with advanced HF, is anemia, when available iron stores are not sufficient for new RBCs production. The general picture is different from either functional or absolute ID what is consistent with suggestions by Ghafourian et al. [48]. We observe a drop in hepatic hepcidin production with concomitant iron deposition in unavailable form of hemosiderin without the presence of inflammation. Progression of HF is driven by the sustained activation of the sympathetic nervous system and RAAS exerting deleterious effects on the heart and the circulation [49]. It was shown that angiotensin II in mice reduced hepatic hepcidin levels, contributing to the alteration of body iron distribution [50]. These data along with our results might suggest that RAAS activation is responsible for decreased production of hepcidin within the liver, connecting one of neurohormonal systems with iron metabolism. Recent papers suggest that ID could be a key factor, not just a “bystander”, in the pathophysiological sequence that leads to the HF progression underlying the interplay between iron metabolism disturbances and neurohormonal activation [51,52].

This work is the first insight into hepatic and cardiac ability to store iron in a porcine HF model. Our approach ensures lack of co-morbidities, concomitant therapy, different genetic background and variable diet, as well as tissue samples accessibility. What is more, the iron metabolism is well balanced in pigs [53]. Our results suggest down-regulation of iron availability in the liver as well as reduced hepatic iron storing ability in HF. In turn, the iron storage capacity in a failing heart is higher than in controls, while myocardial iron stores are reduced. Thus, treatment of HF patients with intravenous iron might replenish myocardial iron stores, enhancing substrate utilization and myocardial energetic which could explain observed improvements in quality of life, exercise capacity, and cardiac function [43,54]. Our results indicate the liver as a main player involved in ID in HF. Paradoxically, during the development of congestive hepatopathy, iron is massively arrested in form of hemosiderin aggregates, while the organism simultaneously occurs systemic iron depletion. This paradox might be a result of a kind of a vicious cycle: (i) HF-induced hypoxia force the organism to produce more RBCs, (ii) RBCs are arrested inside the liver due to the congestion, (iii) iron from congested RBCs is reused to produce more RBC, (iv) the capacity of Kupffer cells and hepatocytes is overwhelmed and iron cumulates in form of hemosiderin and is becoming unavailable for the organism.

Finally, we would like to mention some limitations of the current study. First of all, HF was induced in adult but not in old pigs, and we did not generate a prooxidative and proinflammatory milieu, typical for aged individuals. What is more, working on an animal HF model, we did not have comorbidity, and polypharmacy, typical for HF patients. Secondly, development of severe HF took months not years as in case of human HF patients. We believe that RV pacing in pigs produces a reliable model of chronic HF, mimicking the natural evolution of heart insufficiency, accompanied by compensatory mechanisms.

In conclusion, we used HF porcine model to have insight into hepatic and cardiac ability to store iron. Our results indicate the liver as an important player involved in systemic changes in iron homeostasis in HF. We show that hepatic congestion-induced accumulation of iron in hepatocytes and Kupffer cells make iron no longer available for systemic/cardiac needs in HF. At the same time, in the failing heart, the iron storage reserve is increased. Our findings might be helpful to identify new targets for medical treatment of HF.

## 4. Materials and Methods

### 4.1. Study Approval

All animal experiments were approved by the Bioethical Committee of the Wroclaw University of Environmental and Life Sciences (Approval No 40/2011). All animal procedures were in accordance with the guidelines from Directive 2010/63/EU of the European Parliament on the protection of animals used for scientific purposes and to the Guide for the Care and Use of Laboratory Animals as published by the National Institutes of Health (NIH publication No. 85-23, revised in 1996). All pigs were housed in individual cages under light-controlled conditions and room temperature. Animals were allowed to acclimate 14 days prior to any intervention.

### 4.2. Experimental Design

The study was performed on 32 Polish Large White breed adult pigs (8-month-old siblings, males, 111 ± 19 kg). All procedures and echocardiographic measurements were performed under anesthesia, preceded with food 12-h restriction and 4-h water restriction. Pigs were anesthetized as described in our previous studies [55]. Briefly, animals were premedicated with an intramuscular injection of 1 mg/m^2^ body surface area (BSA) medetomidine hydrochloride (Cepetor, CP-Pharma, Burgdorf, Germany), 5 mg/m^2^ BSA of midazolam (Midanium, WZF Polfa, Warsow, Poland) and 264 mg/m^2^ BSA of ketamine (Bioketan, Vetoquinol Biowet, Gorzów Wlkp, Poland) in a mixing syringe. An ear vein was punctured for the placement of a catheter for an intravenous induction of propofol (Propofol 1% MCT/LCT Fresenius, Fresenius Kabi, Warsow, Poland) at 2–5 mg/kg body weight (BW). Following intubation (8.5 Charriere tubes with blunt-tipped plastic guide wire), anesthesia was maintained by continuous infusion of 1–3 μg/h per kilogram BW fentanyl (Fentanyl WZF, WZF Polfa, Warsow, Poland) and inhalation of isoflurane (1.5–2% vol) (Aerrane, Baxter, Warsaw, Poland). Monitoring of the basal life functions (ECG, end-tidal CO_2_, oxygen saturation, noninvasive blood pressure) was carried out using LIFEPAK 12 Defibrillator/Monitor (Medtronic, Warsow, Poland). Pacemakers were implanted as previously described by this laboratory [8,55]. After a 14-day recovery, the pacemakers were programmed for sequential RV pacing at 170 beat per minute in 26 randomly chosen pigs. A total of 6 sham-operated animals were cared for in an identical fashion with the exception of pacing activation. The control examination was performed monthly and comprised: (1) clinical assessments with an evaluation of HF signs and symptoms [54], (2) transthoracic echocardiography.

Animals developing the consecutive HF stages (mild, moderate and severe) were euthanized. Control animals were euthanized in parallel to HF pigs and were randomly selected for the procedure. Venous blood samples were taken from each animal directly before euthanasia, and complete blood count (Hb, HCT, MCV, MCH, MCHC, RBC, WBC, PLT as well as biochemical blood analysis (ALT, AST, total serum protein, serum albumin, total serum bilirubin, glucose, triglyceride) was performed. Pigs were euthanized with a single intravenous bolus injection of an overdose of sodium pentobarbital (Morbital, Biowet Pulawy, Poland) (≥100 mg/kg), and shortly, an autopsy was performed. Heart and liver weights were determined. Tissue sections from the liver and LV free wall were harvested and frozen in liquid nitrogen. At the same time, separate sections for standard histology were immersed in a 4% buffered formalin (pH 7.2) solution and stored for the further assessments.

### 4.3. Echocardiography

An imaging ultrasound system (Aloka 4000+ with a 3.5 MHz phased array transducer, Aloka Company, Tokio, Japan) was used to carry out transthoracic echocardiography as previously described [17]. LV posterior wall (LVPW) thickening was calculated using the equation = [(LVPWs - LVPWd/LVPWd)] · 100, and expressed as a percentage (LVPWs, LV posterior wall thickness at systole; LVPWd, posterior wall thickness at diastole).

### 4.4. Neurohormonal Activation and Serum Iron Status

Venous blood samples were drawn from each pig directly before euthanasia and immediately processed and stored as serum and plasma samples at −80 °C until further analyses. Plasma renin activity (PRA, ng/mL/h of a generated angiotensin I) was measured using an ELISA test (DiaSource, Gentaur, Sopot, Poland) following the manufacturer’s instructions. Serum B-type natriuretic peptide (BNP, ng/mL) was assessed at a 1:5 dilution using a Peptide Enzyme Immunoassay (EIA) Kit (Bachem, Bubendorf, Switzerland) according to the manufacturer’s recommendations. Cortisol and aldosterone were measured in serum using an ELISA Kit (Cayman Chemical, Biokom, Poland and DRG, Marburg, Germany;accordingly) as described by Zacharski et al. [55]. Plasma adrenaline and noradrenaline were assayed using CatCombi ELISA (Tecan Group Ltd., Mannendorf, Switzerland) as described by Tomaszek et al. [56]. All measurements were performed in duplicates.

Serum iron concentrations (µg/dL) and Total Iron Binding Capacity (TIBC) (µg/dL) were assessed using a substrate method with Ferene S (Thermo Fisher Scientific, Warsaw, Poland). Transferrin saturation (TSAT) was calculated as a ratio between serum iron (µg/dL) and TIBC (µg/dL), multiplied by 100 and expressed as a percentage (these values were measured in 19 individuals). Serum ferritin was measured by means of commercially available ELISA kit (Abcam, Symbios, Straszyn, Poland) following the recommendations of the manufacturer. Serum erythropoietin (EPO) was assessed by two ELISA kits (Abnova, , Prospecta, Warsaw, Poland; R&D Systems, Biotechne, Warsaw, Poland), specific for human EPO, as a reliable ELISA kit specific for porcine EPO was not available.

### 4.5. Cytokine Measurement

Liver samples (about 30 mg) were prepared as previously described [57]. Hepatic amount of IL-1β and IL-6 was measured by ELISA (R&D Systems, Biotechne, Warsaw, Poland). Briefly, precoated wells were incubated overnight with 100 μL of homogenate diluted 1:25 in Reagent Diluent, followed by washing 4 times with wash buffer. Wells were then incubated with appropriate biotinylated detection antibody and washed again before incubation with streptavidin conjugated to horseradish-peroxidase. After incubation with a substrate, the plates were read by spectrophotometry at 450 nm, in accordance with the manufacturer’s specifications. All samples were measured in duplicate. ELISA results in each sample were normalized using the total protein concentrations and reported as ng/mg protein.

Fresh-frozen serum samples (−80 °C) obtained directly before euthanasia were used for cytokine measurements (IL-1β, IL-6, TNF-α) with Quantikine ELISA kit (R&D Systems, Biotechne, Warsaw, Poland) according to the manufacturers’ instructions. The results were reported as pg/mL.

### 4.6. Histological Analyses

Sections from the left ventricle and liver of control and TIC pigs were fixed in 4% buffered formalin (pH 7.2) and embedded in paraffin wax. The 3-μm transverse sections were stained with hematoxylin and eosin (H&E staining). Images were analyzed in a Nikon Eclipse 80i microscope (Nikon Instruments Inc., Amsterdam, Netherlands). A scoring system from − to +++ was used, correlating to, respectively, no lesion (−), mild lesion (+), moderate lesion (++), and severe lesion (+++).

### 4.7. Non-Heme Iron Histochemistry

The non-heme iron in the liver, and myocardial sections was visualized by the Prussian Blue Reaction (Fe^3+^ detection, Mallory’s Method) and Turnbull’s Blue Reaction (Fe^2+^ detection), respectively [58,59]. After deparaffinization in xylene and rehydration through graded ethanol, tissue sections (LV and liver) were incubated for 30 min in 5% potassium ferrocyanide in aqueous hydrochloric acid (5%) (Fe^3+^ detection) or in potassium ferricyanide staining solution (1% potassium ferricyanide in 0.5% HCl) (Fe^2+^ detection). Nuclear fast red (Sigma-Aldrich, Poznań, Poland) was used as a counterstain [60]. Deposits of iron were stained as blue by both Prussian Blue or Turnbull’s Blue reaction, while cytoplasm and cellular nucleus were stained as pink and red, respectively. With the Prussian blue stain, ferritin is seen as a faint blue cytoplasmic blush. Hemosiderin appears as coarse blue granules [61]. Images were analyzed with a Nikon Eclipse 80i microscope (Nikon Instruments Inc.). Evaluation of iron staining (Fe^2+^ and Fe^3+^) included the following categories: negative (−), weak (+), moderate (++), and strong (+++).

### 4.8. Immunohistochemistry

The expression of the ferritin (light and heavy chain) in the sections of porcine LV myocardium and liver was determined using immunohistochemical (IHC) staining with a rabbit polyclonal anti-ferritin light chain and anti-ferritin heavy chain antibody (Abcam, Symbios, Straszyn, Poland). Tissue sections were prepared as previously described [57]. Immunohistochemical reactions were performed using antibodies detecting FTL (1:250) and FTH (1:100) over night at 4 °C. Then, slides were incubated with secondary biotinylated antibody (15 min, room temperature) and a streptavidin–peroxidase complex (15 min, room temperature) (LSAB, HRP; Dako, Warsaw, Poland). NovaRed (Vector Laboratories, Biokom, Warsaw, Poland) was used as a chromogen (10 min, room temperature). All the sections were counterstained with Meyer’s hematoxylin (Sigma-Aldrich, Poznań, Poland). The negative control was performed by omitting the primary antibody. Images were analyzed using a Nikon Eclipse 80i microscope (Nikon Instruments Inc., Amsterdam, Netherlands). Evaluation of IHC expression included the following categories: negative (−), weak (+), moderate (++), and strong (+++).

### 4.9. Ferritin Isolation

Isolation procedures of ferritin have not changed since its crystallization in 1937, they all involve the homogenization of the tissue and heating the homogenate to 80 °C [62]. The heating step does not damage the ferritin molecule, which remains in the solution, whereas other proteins (including heme) are denatured (coagulated) and removed by centrifugation [16,63]. What is more, the ferritin iron core is insensitive to heat treatment [64].

Ferritin was prepared from the tissues (liver and left ventricle) using the method described by Miyazaki et al. [36]. Briefly, each tissue sample (100 mg) was homogenized in four volumes (*w*/*v*) of distilled water. The homogenates were then incubated at 80 °C for 10 min and centrifuged for 15 min at 14,000× *g* at 4 °C. The ferritin enriched supernatants were collected and analyzed further. Purity of the ferritin protein in the supernatant (ferritin rich fraction) is evaluated to be 72–75% [36].

### 4.10. SDS-PAGE, Prussian Blue Reaction, Western Blotting

SDS-PAGE was conducted according to the method of Laemmli [65], with 8% acrylamide separating gels and 4% stacking gels, each containing 1% SDS. The ferritin-enriched supernatants (10 μL) were mixed with a non-reducing sample buffer (Pierce), followed by incubation for 10 min at 40 °C. For detection of iron-loaded ferritin by Prussian Blue staining, the reducing agents in the sample buffer had to be omitted in order to avoid Fe^3+^ reduction [66]. Gels were stained with a mixture of 5% ferrocyanide [K_4_Fe(CN)_6_] and 5% hydrochloric acid (1:1, *v*/*v*) prepared immediately before use. The iron-loaded ferritin was visible as a blue band on the top of the gel. The intensities of the bands were measured by densitometric scanning using the ChemiDoc MP Imaging System and Image LabTMSoftware v.6.0 (BioRad, Warsaw, Poland). One of the control pigs was chosen as an internal control used to compare Fe^3+^ staining on every gel. The intensity of the target band signal in the individual sample was divided by the intensity of the target band in the control. The resulting ratios (relative intensities), given as fold change, were used to compare Fe^3+^ staining of iron-loaded ferritin levels across analyzed samples. Each sample was analyzed in triplicate.

We used SDS-PAGE gels prepared in the same way to confirm that ferritin-enriched supernatants contain complex ferritin (high-molecular-weight oligomeric cage) assembled from ferritin L- and H-chains. SDS-PAGE gels were wet-transferred to a PVDF membrane (Millipore, Poland). The membrane was treated with the Quentix Signal Enhancer (Pierce, Thermofisher Scientific, Warsaw, Poland), blocked for 1 h with 5% skimmed milk in the PBS containing 0.5% (*v*/*v*) Triton X-100 (Sigma-Aldrich), and incubated overnight with rabbit antibodies against FTH (1:500) (Abcam, Symbios, Straszyn, Poland) or against FTL (1:1000) (Abcam, Symbios, Straszyn, Poland). Bands were detected using the SuperSignal West Femto ECL substrate (Pierce, Thermofisher Scientific, Warsaw, Poland). The intensities of the bands were determined by densitometric scanning using the ChemiDoc MP Imaging System and Image LabTMSoftware v.6.0 (BioRad, Warsaw, Poland). One of the control pigs was chosen as the internal control and was used to compare the amount of assembled ferritin (FTL and FTH) on every blot. The intensity of the target band signal in individual sample was divided by the intensity of the target band in the control. The resulting ratios (relative intensities), given as fold change were used to compare assembled ferritin levels across analyzed samples. Each sample was analyzed in triplicate.

### 4.11. RNA Isolation and Real-Time PCR

Porcine liver tissue samples (30 mg) were homogenized with a TissueRuptor (Qiagen, Wrocław, Poland). Total RNA was purified with the RNeasy Fibrous Tissue Mini Kit (Qiagen, Wrocław, Poland). First-strand cDNA was synthesized using a SuperScript III First-Strand Synthesis System with an oligo(dT)20 primer (Invitrogen, Thermofisher Scientific, Warsaw, Poland). Then, the relative amounts of porcine hepcidin (*HAMP*) in hepatic samples were determined by reverse transcription quantitative polymerase chain reaction (RT-qPCR) using the CFX ConnectTM Real-Time PCR Detection System (Bio-Rad, Warsaw, Poland) with the SSoFast Eva Green Supermix (Bio-Rad, Warsaw, Poland). All samples were performed in triplicate. Transcript levels were normalized to glyceraldehyde-3-phosphate dehydrogenase (*GAPDH*) using the ΔΔCT method. mRNA expression was presented in arbitrary units (AU), where the sample from the liver from one of the control pigs was chosen as the calibrator, and its mRNA expression was considered as 1. Primer sequences used in this manuscript are displayed in Supplementary Table S6.

### 4.12. Aconitase Activity

Bioxytech Aconitase-340 kit (OxisResearch, Foster City, CA, USA) was used to measure aconitase activity in porcine tissues as previously described [57].

### 4.13. Measurement of Malonyldialdehyde Level

The BIOXYTECH MDA-586 kit (OxisResearch, Foster City, CA, USA) was used to measure the MDA level in the serum, liver and LV homogenates as previously described [57].

### 4.14. Statistical Analysis

Values are expressed as mean with standard deviation (SD) unless otherwise indicated. For all correlation analyses, Spearman’s rank correlation coefficients were used. Relationships between echocardiographic, hematological and biochemical parameters, indices of iron, inflammatory as well as oxidative stress status, and HF severity were calculated using Spearman’s rank correlation coefficients. The Mann–Whitney U test was used to assess the statistical differences in mean values of echocardiographic data, biochemical data, iron staining and immunoblotting data between controls and subsequent HF groups. All statistical analyses were performed using Statistica for Windows (Statsoft, Cracov, Poland), software version 12. A *p* value less than 0.05 was considered statistically significant.

## Figures and Tables

**Table 1:** Hematological parameters and indices of iron status in sham-operated and TIC pigs (mild, moderate, and severe HF groups).

	Controls (*n* = 6)	Mild HF (*n* = 9)	Moderate HF (*n* = 9)	Severe HF (*n* = 8)	Spearman Correlatory Rank Coefficients R with *p*, for All Animals
Hematological parameters					
RBC (×10^12^/L)	5.62 ± 0.51	5.88 ± 0.47	5.86 ± 0.83	6.72 ± 1.09 *	R = 0.38, *p* = 0.03
R-RBC (%)	86.4 ± 7.5	96.2 ± 6.3 *	99.2 ± 7.3 **	110.2 ± 19 *	R = 0.57, *p* = 0.006
Hb (mmol/L)	5.96 ± 0.40	5.81 ± 0.39	5.96 ± 0.82	7.09 ± 1.48	R = 0.33, *p* = 0.07
R-Hb (%)	92.2 ± 6.3	98.6 ± 5.8	101.1 ± 7.3 *	120.1 ± 23 *	R = 0.67, *p* = 0.00007
HCT (%)	30.9 ± 1.6	31.0 ± 2.2	32.1 ± 3.9	37.4 ± 7.9	R = 0.39, *p* = 0.03
R-HCT (%)	92.3 ± 7.9	97.0 ± 6.2	103.5 ± 8.4 *	122.7 ± 27 *	R = 0.62, *p* = 0.0002
WBC (×10^9^/L)	15.90 ± 6.13	14.00 ± 3.44	11.70 ± 2.29	9.00 ± 1.26 **	R = −0.63, *p* = 0.0001
PLT (×10^9^/L)	303 ± 103	235 ± 46	188 ± 60 *	219 ± 48	R = −0.35, *p* = 0.05
MCV (fL)	54.5 ± 2.43	52.8 ± 1.98	53.7 ± 2.61	54.4 ± 2.88	R = 0.02, *p* = 0.89
MCH (fmol)	1.03 ± 0.06	0.99 ± 0.05	1.01 ± 0.08	1.04 ± 0.07	R = 0.09, *p* = 0.60
MCHC (mmol/L)	18.8 ± 0.9	18.7 ± 0.8	18.7 ± 0.6	18.9 ± 0.7	R = −0.02, *p* = 0.87
Iron status					
Serum iron (μg/dL)	126 ± 21	100 ± 23	106 ± 20	74 ± 23 **	R = −0.52, *p* = 0.002
TSAT (%) ^#^	39.1 ± 6,9	31.9 ± 7.9	30.1 ± 13	22.0 ± 7.5 *	R = −0.57, *p* = 0.01
TIBC (μg/dL) ^#^	343 ± 29	315 ± 42	416 ± 103	412 ± 83	R = 0.44, *p* = 0.06
Serum ferritin (ng/mL)	1.98 ± 2.17	1.88 ± 3.04	0.94 ± 0.78	1.54 ± 0.46	R = 0.16, *p* = 0.38

RBC, red blood cell count; R-RBC, relative change in RBC during the experiment time (RBC in end-point/RBC in t = O)∗100%); Hb, hemoglobin concentration; R-Hb, relative change in Hb during the experiment time (Hb in end-point/Hb in t = O)∗100%); HCT, hematocrit; R-HCT; relative change in HCT during the experiment time (HCT in end-point/HCT in t = O)∗100%); WBC, White Blood Cell count; PLT, platelet count; MCV, mean corpuscular volume; MCH, mean corpuscular hemoglobin; MCHC, mean corpuscular hemoglobin concentration; TSAT, transferrin saturation; TIBC, total iron binding capacity; ^#^ these values were measured in 19 individuals, controls (*n* = 5), mild (*n* = 4), moderate (*n* = 6), and severe (*n* = 4) HF. Values are described as means ± standard deviation. Statistical significance was determined by the Mann–Whitney U test (* *p* < 0.05 vs. control group; ** *p* < 0.01 vs. control group). Relationships between analyzed parameters and HF severity (0—controls, 1—mild HF, 2—moderate HF, 3—severe HF) were tested using Spearman’s rank correlation coefficients.

**Table 2:** Biochemical parameters and indices of inflammatory and oxidative stress status in sham-operated and HF pigs (mild, moderate, and severe HF groups).

	Controls (*n* = 6)	Mild HF (*n* = 9)	Moderate HF (*n* = 9)	Severe HF (*n* = 8)	Spearman Correlatory Rank Coefficients R with *p*, for All Animals
Biochemical parameters					
Serum albumin (g/L)	36.5 ± 3.0	34.7 ± 2.7	37.0 ± 2.6	31.8 ± 3.1	R = −0.27, *p* = 0.17
Total serum protein (g/L)	61.5 ± 5.6	57.2 ± 3.9	56.7 ± 4.4	59.6 ± 7.0	R = −0.02, *p* = 0.9
Total serum bilirubin (μmol/L)	1.62 ± 1.32	1.83 ± 1.62	2.47 ± 2.45	4.01 ± 5.21	R = 0.29, *p* = 0.11
AST (U/L)	21.33 ± 11.08	21.56 ± 8.99	29.38 ± 12.25	35.33 ± 12.25	R = 0.42, *p* = 0.02
ALT (U/L)	51.3 ± 17.4	41.6 ± 5.4	39.5 ± 21.1	32.3 ± 15.9	R = −0.32, *p* = 0.08
AST/ALT ratio	0.43 ± 0.21	0.52 ± 0.22	1.04 ± 0.95	1.88 ± 1.72 *	R = 0.54, *p* = 0.002
Glucose (mmol/L)	4.73 ± 0.71	5.39 ± 0.63	5.39 ± 0.91	5.86 ± 0.97 *	R = 0.37, *p* = 0.03
Triglyceride (mmole/L)	0.27 ± 0.07	0.29 ± 0.09	0.36 ± 0.08	0.43 ± 0.13	R = 0.47, *p* = 0.006
Inflammatory and oxidative stress status					
Serum IL-1β (pg/mL)	2.53 ± 2.93	4.04 ± 3.34	8.96 ± 9.7	5.09 ± 4.37	R = 0.24, *p* = 0.19
Serum IL-6 (pg/mL)	1.61 ± 2.15	6.02 ± 4.78	7.55 ± 7.97	7.89 ± 7.07 *	R = 0.31, *p* = 0.08
Serum TNF-α (pg/mL)	56.2 ± 27.8	73.2 ± 30.7	78.1 ± 46.1	64.4 ± 33.1	R = 0.07, *p* = 0.69
Serum MDA (μmol/L)	6.79 ± 2.42	6.19 ± 1.58	8.92 ± 6.70	8.46 ± 2.09	R = 0.36, *p* = 0.04
Liver aconitase activity in mitochondrial fraction (mU/mg of protein)	2.42 ± 1.03	3.59 ± 0.83	1.68 ± 1.10	1.39 ± 0.99	R = −0.50, *p* = 0.004
Liver aconitase activity in cytoplasmic fraction (mU/mg of protein)	7.08 ± 3.69	10.03 ± 3.50	6.90 ± 2.07	3.92 ± 3.00	R = −0.40, *p* = 0.02
Liver MDA (nmol/mg of tissue)	0.07 ± 0.06	0.09 ± 0.08	0.07 ± 0.05	0.1 ± 0.06	R = 0.12, *p* = 0.50
LV aconitase activity in mitochondrial fraction (mU/mg of protein)	0.36 ± 0.56	0.36 ± 0.30	0.99 ± 1.56	0.52 ± 1.05	R = −0.05, *p* = 0.77
LV aconitase activity in cytoplasmic fraction (mU/mg of protein)	0.21 ± 0.17	0.09 ± 0.09	0.28 ± 0.34	0.44 ± 0.42	R = 0.33, *p* = 0.07
LV MDA (nmol/mg of tissue)	0.05 ± 0.02	0.04 ± 0.01	0.03 ± 0.01	0.04 ± 0.01	R = −0.22, *p* = 0.30

AST, aspartate aminotransferase; ALT, alanine aminotransferase; MDA, malonyldialdehyde; LV; left ventricle. Values are described as means ± standard deviation. Statistical significance was determined by the Mann–Whitney U test (* *p* < 0.05 vs. control group). Relationships between examined parameters and HF severity (0—controls, 1—mild HF, 2—moderate HF, 3—severe HF) were tested using Spearman’s rank correlation coefficients.

## Data Availability

The data presented in this study are available in the published article.

## Supplementary Materials

The following are available online at https://www.mdpi.com/article/10.3390/ijms23031026/s1.
