# Supplementary material for: Hemosiderin Accumulation in Liver Decreases Iron Availability in Tachycardia-Induced Porcine Congestive Heart Failure Model
Source: Int J Mol Sci. 2022 Jan 18;23(3):1026. doi: 10.3390/ijms23031026 (PMC8834801; doi:10.3390/ijms23031026)
Supplement: Supplementary file 1 [file ijms-23-01026-s001.zip › ijms-1520237-supplementary.pdf]

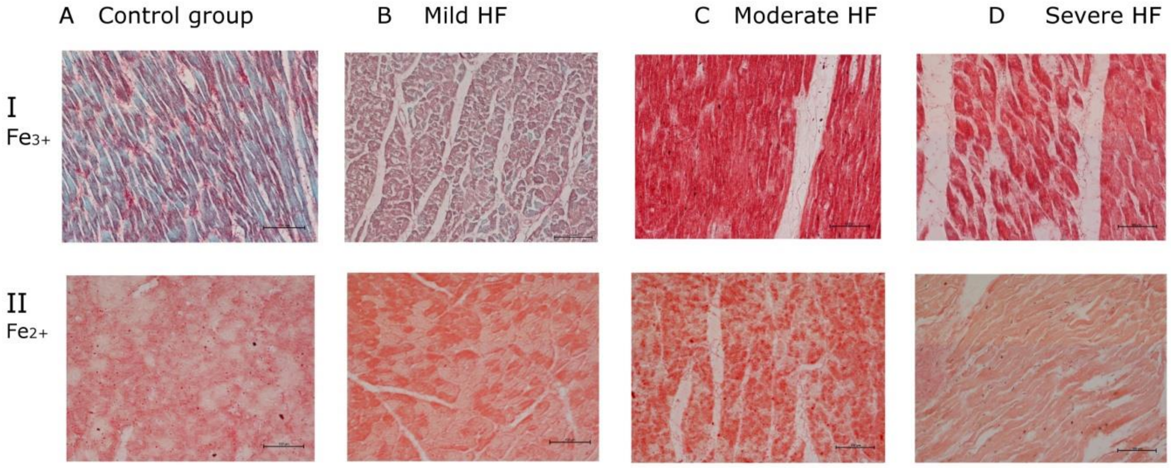

Supplementary Figure S1. Histochemical detection of tissue Fe^{3+} by Prussian Blue reaction (panel I), and Fe^{2+} by Turnbull's Blue reaction (panel II) in representative LV sections from control group (A), pigs with mild HF (B), moderate HF (C), and severe HF (D). 200×original magnification

Supplementary Table S1: Biometric parameters, echocardiographic parameters reflecting the morphology and functioning of left ventricle, and neurohormonal activation in sham-operated and HF pigs (mild, moderate, and severe HF groups).

Variables, units	controls (n=6)	mild HF (n=9)	moderate HF (n=9)	severe HF (n=8)	Spearman correlatory rank coefficients R with *P*, for all animals
Biometric parameters					
heart wt/body wt (g/kg)	3.8 ± 0.6	5.1 ± 1.2*	7.2 ± 2.3*	6.1 ± 2.3*	R=0.56, *P*=0.002
liver wt/body wt (g/kg)	19.0 ± 4.3	29.4 ± 10.1**	22.9 ± 3.7	41.9 ± 7.9**	R=0.70, *P*=0.00004
Echocardiographic parameters					
LVEF (%)	53 ± 8	43 ± 12	29 ± 13**	25 ± 8**	R=-0.70, *P*=0.000006
LVIDd (cm)	5.92 ± 0.40	6.73 ± 0.43**	7.57 ± 0.37**	7.38± 0.35**	R=0.74, *P*=0.000001
LVIDs (cm)	4.50 ± 0.31	5.21 ± 0.46**	6.46 ± 0.50**	6.46 ± 0.43**	R=0.81, *P*=0.000001
LVEDV (mL)	176 ± 27	237 ± 30**	305 ± 32**	284±32**	R=0.73, *P*=0.000002
LVESV (mL)	93 ± 15	132 ± 28**	214 ± 34**	214±34**	R=0.80, *P*=0.000001
LVPW thickening (%)	52 ± 8	53 ± 10	42 ± 26	19±14**	R=-0.65, *P*=0.00005
Neurohormonal activation					
BNP (ng/mL)	0.26 ± 0.23	0.27 ± 0.16	0.59 ± 0.46	0.72 ± 0.32*	R=0.52, *P*=0.003

PRA (ng/mL/h)	0.51 ± 0.60	1.02 ± 0.86	1.56 ± 1.54	8.08 ± 6.45**	R=0.62, *P*=0.0001
ADR (nmol/L)[#]	0.74 ± 0.80	0.57 ± 0.85	2.11 ± 2.41	4.18 ± 2.58	R=0.45, *P*=0.04
NOR (nmol/L)[#]	1.23 ± 0.34	0.71 ± 0.37*	5.06 ± 3.40*	4.65 ± 3.74	R=0.41, *P*=0.06
Aldosterone (pg/ml)	548.9±163.2	769.3±315.1	777.9±277.5	1554±1132	R=0.54, *P*=0.001
Cortisol (ng/ml)	40 ± 13	47 ± 15	52 ± 17	61 ± 32	R=0.40, *P*=0.025

wt, weight; LVEF, LV ejection fraction; LVIDd, LV internal dimension at end diastole; LVEDV, LV end-diastolic volume; LVESV, LV end-systolic volume; LVPW, LV posterior wall; LVPW thickening = [(LVPWs – LVPWd)/LVPWd)] x 100% (LVPWs, LV posterior wall thickness at systole; LVPWd, LV posterior wall thickness at diastole); BNP, B-type natriuretic peptide; PRA, plasma renin activity; ADR, adrenaline; NOR, noradrenaline. [#] these values were measured in 21 individuals, controls (n=5), mild (n=6), moderate (n=6), and severe (n=4) HF.

All Values are described as means ± standard deviation. Statistical significance was determined by the Mann-Whitney U test (* *P* <0.05 vs. control group; ** *P* <0.01 vs. control group).

Relationships between examined parameters and HF severity were analyzed using Spearman's rank correlation.

Supplementary Table S2: Relationships between hematological parameters, indices of iron status, and echocardiographic parameters, as well as the measures of neurohormonal activation in all pigs.

	RBC ($\times 10^{12}$/L)	R-RBC (%)	Hb (mmol/L)	R-Hb (%)	HCT (%)	R-HCT (%)	WBC ($\times 10^{9}$/L)	PLT ($\times 10^{9}$/L)	Serum iron (μg/dL)	TSAT (%)	TIBC (μg/dL)
heart wt/body wt (g/kg)	ns	ns	ns	ns	ns	ns	ns	- 0.41<br>*0.03*	ns	ns	ns
liver wt/body wt (g/kg)	0.58<br>*0.001*	0.55<br>*0.002*	0.49<br>*0.008*	0.56<br>*0.002*	0.56<br>*0.002*	0.40<br>*0.04*	- 0.49<br>*0.008*	ns	- 0.62<br>*0.0005*	-0,58<br>*0,02*	ns
LVEF (%)	ns	ns	ns	ns	ns	ns	0.51<br>*0.002*	0.54<br>*0.002*	0.58<br>*0.0004*	0.62<br>*0.004*	ns
LVIDd (cm)	ns	0.36<br>*0.04*	ns	0.45<br>*0.01*	ns	0.38<br>*0.03*	ns	ns	- 0.36<br>*0.04*	- 0.49<br>*0.03*	ns
LVIDs (cm)	ns	0.35<br>*0.04*	ns	0.47<br>*0.009*	ns	0.38<br>*0.04*	- 0.49<br>*0.004*	- 0.49<br>*0.005*	- 0.50<br>*0.003*	- 0.70<br>*0.0066*	0.47<br>*0.04*
LVEDV (mL)	ns	0.36<br>*0.04*	ns	0.45<br>*0.01*	ns	0.37<br>*0.04*	ns	ns	ns	- 0.48<br>*0.04*	ns
LVESV (mL)	ns	ns	ns	0.46<br>*0.003*	ns	0.36<br>*0.04*	- 0.50<br>*0.004*	- 0.51<br>*0.004*	- 0.49<br>*0.004*	- 0.68<br>*0.001*	0.49<br>*0.03*
LVPW thickening (%)	- 0.46<br>*0.007*	ns	- 0.44<br>*0.01*	- 0.44<br>*0.02*	ns	ns	0.60<br>*0.0007*	0.49<br>*0.005*	0.51<br>*0.003*	0.76<br>*0.0001*	- 0.49<br>*0.03*
BNP (ng/mL)	ns	ns	0.40<br>*0.03*	ns	0.41<br>*0.03*	0.37<br>*0.05*	ns	- 0.39<br>*0.03*	ns	ns	ns
PRA (ng/mL/h)	0.51	0.51	0.43	0.58	0.61	0.46	- 0,42	ns	- 0.39	- 0.58	ns

	0.003	*0.003*	*0.02*	*0.0008*	*0.0003*	*0.008*	*0,02*		*0.03*	*0.01*	
Aldosterone (pg/ml)	0.41<br>*0.02*	0.54<br>*0.001*	ns	0.53<br>*0.003*	0.51<br>*0.003*	0.45<br>*0.01*	-0.42<br>*0.02*	ns	ns	ns	0.47<br>*0.04*
ADR (nmol/L)	ns	ns	ns	ns	ns	ns	-0.66<br>*0.001*	-0.58<br>*0.007*	ns	-0.54<br>*0.02*	0.53<br>*0.02*
NOR (nmol/L)	ns	ns	ns	ns	ns	ns	-0.53<br>*0.01*	ns	*ns*	*ns*	-0.66<br>*0.002*
Cortisol (ng/ml)	ns	ns	ns	ns	ns	0.44<br>*0,02*	- 0,38<br>*0,04*	ns	- 0.45<br>*0.01*	- 0.58<br>*0.01*	0.59<br>*0.008*

wt, weight; LVEF, LV ejection fraction; LVIDd, LV internal dimension at end diastole; LVEDV, LV end-diastolic volume; LVESV, LV end-systolic volume; LVPW, LV posterior wall; LVPW thickening = [(LVPWs – LVPWd)/ LVPWd)] x 100% (LVPWs, LV posterior wall thickness at systole; LVPWd, LV posterior wall thickness at diastole); BNP, B-type natriuretic peptide; PRA, plasma renin activity; RBC, Red Blood Cell count, R-RBC, relative change in RBC during the experiment time (RBC in end-point/RBC in t=O)*100%); Hb, hemoglobin concentration; R-Hb, relative change in Hb during the experiment time (Hb in end-point/Hb in t=O)*100%); HCT, hematocrit; R-HCT; relative change in HCT during the experiment time (HCT in end-point/HCT in t=O)*100%); WBC, white blood cell count; PLT, platelet count; TSAT, transferrin saturation; TIBC, total iron binding capacity; ADR, adrenaline; NOR, noradrenaline; ns, non-significant.

For all correlatory analyses Spearman's rank correlatory coefficients were used. This table presents R value, and the *P* statistics (in italics) for each significant correlation.

Supplementary Table S3: Relationships between hematological parameters, indices of iron status, echocardiographic parameters, the measures of neurohormonal activation, and chosen biochemical parameters, and relative ferritin-bound Fe^{3+}, assembled ferritin (FTL and FTH) relative level in liver and LV in all pigs.

	**Serum albumin (g/L)**	**AST/ALT**	**Glucose (mmol/L)**	**Liver relative hepcidin expression**	**Liver relative ferritin-bound Fe^{3+} level**	**Liver relative FTL level**	**Liver relative FTH level**	**LV relative ferritin-bound Fe^{3+} level**	**LV relative FTL level**	**LV relative FTH level**
RBC (×10[12]/L)	ns	0.64<br>*0.0002*	0,37<br>*0,04*	- 0.42<br>*0.02*	- 0.39<br>*0.03*	ns	ns	ns	ns	ns
R-RBC (%)	ns	0.59<br>*0.0007*	0,38<br>*0,03*	- 0.62<br>*0.0003*	ns	- 0.44<br>*0.01*	ns	ns	ns	ns
Hb (mmol/L)	ns	0.55<br>*0.002*	0,36<br>*0,05*	- 0.44<br>*0.02*	ns	ns	ns	ns	ns	ns
R-Hb (%)	ns	0.61<br>*0.0007*	0,37<br>*0,05*	- 0.67<br>*0.0001*	- 0.38<br>*0.04*	- 0.44<br>*0.02*	- 0.40<br>*0.03*	ns	0.52<br>*0.005*	ns

HCT (%)	ns	0.64<br>*0.0002*	0,44<br>*0,01*	- 0.52<br>*0.003*	- 0.40<br>*0.03*	- 0.41<br>*0.02*	ns	ns	ns	ns
R-HCT (%)	ns	0.47<br>*0.01*	ns	- 0.65<br>*0.0002*	ns	- 0.38<br>*0.04*	- 0.43<br>*0.02*	ns	0.48<br>*0.008*	ns
WBC (×10⁹/L)	ns	- 0.41<br>*0.03*	ns	ns	ns	ns	ns	0.49<br>*0.005*	- 0.37<br>*0.04*	-0.41<br>*0,02*
PLT (×10⁹/L)	ns	- 0.49<br>*0.006*	- 0,37<br>*0,04*	ns	ns	ns	ns	0.43<br>*0.02*	ns	ns
Serum iron (µg/dL)	0.38<br>*0.05*	ns	- 0.46<br>*0.007*	ns	0.62<br>*0.0002*	0.46<br>*0.01*	ns	ns	ns	ns
TSAT (%)	ns	- 0.53<br>*0.02*	- 0,49<br>*0.03*	ns	0.65<br>*0.002*	ns	ns	ns	ns	ns
TIBC (µg/dL)	0.64<br>*0.007*	0.73<br>*0.0006*	ns	ns	ns	ns	ns	ns	ns	0.62<br>*0.005*
heart wt/body wt (g/kg)	- 0.53<br>*0.007*	ns	ns	ns	ns	ns	ns	- 0.69<br>*0.00007*	ns	ns
liver wt/body wt (g/kg)	0.58<br>*0.002*	0.45<br>*0.02*	ns	- 0.62<br>*0.0007*	-0.58<br>*0.001*	- 0.62<br>*0.00006*	ns	ns	0.36<br>*0.06*	ns
LVEF (%)	ns	-0.47<br>*0.008*	ns	ns	0.46<br>*0.008*	ns	0.36<br>*0.04*	0.57<br>*0.0008*	ns	ns
LVIDd (cm)	ns	ns	ns	ns	- 0.63<br>*0.0001*	- 0.37<br>*0.04*	- 0.37<br>*0.04*	- 0.57<br>*0.0007*	0,42<br>*0,02*	0.54<br>*0.002*
LVIDs (cm)	ns	0.49<br>*0.006*	ns	ns	ns	- 0.39<br>*0.03*	- 0.41<br>*0.02*	- 0.63<br>*0.0001*	0.44<br>*0.01*	0.38<br>*0.03*
LVEDV (mL)	ns	ns	ns	ns	ns	- 0,36<br>*0,05*	0.35<br>*0.05*	- 0,57<br>*0.0007*	0.41<br>*0,02*	0.54<br>*0.002*
LVESV (mL)	ns	0.45<br>*0.01*	ns	ns	ns	- 0.38<br>*0.03*	- 0.41<br>*0.02*	- 0.64<br>*0.00008*	0.44<br>*0.01*	0.38<br>*0.03*
LVPW thickening (%)	ns	- 0.56<br>*0.001*	- 0,41<br>*0,02*	ns	0.46<br>*0.01*	0.46<br>*0.01*	0.52<br>*0.002*	0.46<br>*0.01*	ns	ns
BNP (ng/mL)	ns	ns	0,39<br>*0,03*	ns	- 0.44<br>*0.02*	- 0,61<br>*0.005*	- 0.41<br>*0.03*	- 0.54<br>*0.002*	ns	ns
PRA (ng/mL/h)	- 0,38<br>*0.04*	0.65<br>*0.0001*	0,39<br>*0,03*	-0.65<br>*0.0001*	- 0.58<br>*0.0006*	- 0.46<br>*0.009*	- 0.36<br>*0.04*	ns	ns	ns
Aldosterone (pg/ml)	ns	0.64<br>*0.0002*	ns	-0.62<br>*0.001*	ns	ns	ns	ns	ns	ns
ADR (nmol/L)	ns	0.66<br>*0.001*	ns	ns	ns	ns	ns	ns	ns	ns
NOR (nmol/L)	ns	0.56<br>*0.009*	ns	ns	ns	ns	-0.54<br>*0.01*	ns	ns	ns
Cortisol (ng/ml)	ns	0.58<br>*0.001*	ns	ns	ns	ns	ns	ns	ns	ns

RBC, Red Blood Cell count; R-RBC, relative change in RBC during the experiment time (RBC in end-point/RBC in t=O)*100%); Hb, hemoglobin concentration; R-Hb, relative change in Hb during the experiment time (Hb in end-point/Hb in t=O)*100%); HCT, hematocrit; R-HCT, relative change in HCT during the experiment time (HCT in end-point/HCT in t=O)*100%); WBC, White Blood Cell count; PLT, platelet count, TSAT, transferrin saturation; TIBC, total iron binding capacity; wt, weight; LVEF, LV ejection fraction; LVIDd, LV internal dimension at end diastole; LVEDV, LV end-diastolic volume; LVESV, LV end-systolic volume; LVPW, LV posterior wall; LVPW thickening = [(LVPWs – LVPWd)/ LVPWd)] x 100%, and expressed as a percentage (LVPWs, LV posterior wall thickness at systole; LVPWd, LV posterior wall thickness at diastole); BNP, B-type natriuretic peptide; PRA, plasma renin activity; AST, alanine aminotransferase; ALT, aspartate aminotransferase; ADR, adrenaline; NOR, noradrenaline; ns, non-significant.

For all correlatory analyses Spearman's rank correlatory coefficients were used. This table presents R value, and the *P* statistics (in italics) for each significant correlation.

Supplementary Table S4: Histopathological findings after Turnbull's Blue (Fe^{2+}), Prussian Blue (Fe^{3+}), and H&E staining's in hepatic lobule (liver sections) from sham-operated and HF pigs (mild, moderate, and severe HF groups).

		Control	Mild HF	Moderate HF	Severe HF
Fe^{2+} (Turnbull's staining)	Kupffer cells	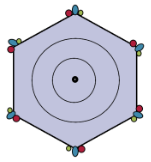	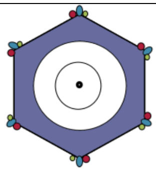	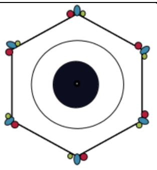	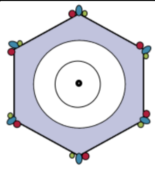
	Kupffer cells – Fe^{2+}aggregates	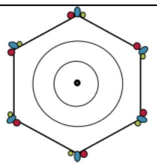	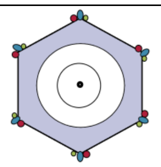	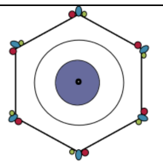	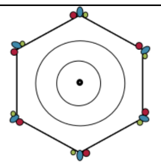
	hepatocytes - diffuse, cytoplasmatic staining	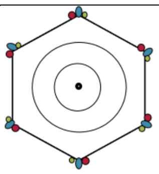	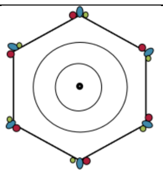	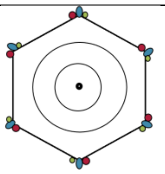	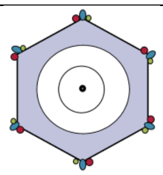
	hepatocytes - Fe^{2+}aggregates	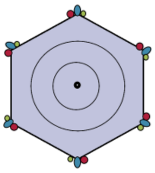	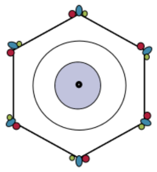	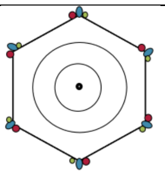	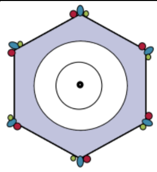
Fe^{3+} (Prussian Blue staining)	Kupffer cells	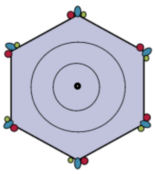	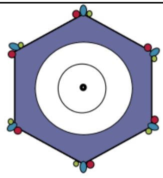	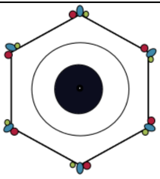	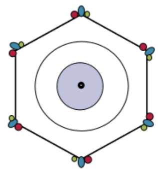
	Kupffer cells – Fe^{3+}aggregates	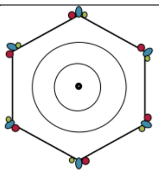	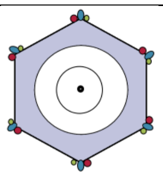	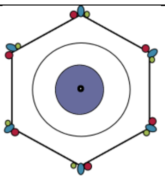	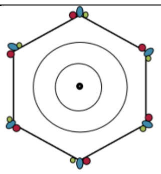
	hepatocytes - diffuse cytoplasmatic staining	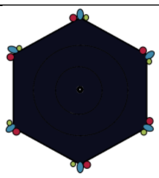	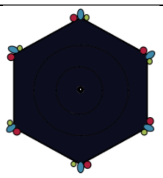	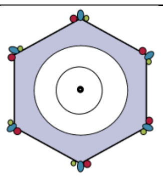	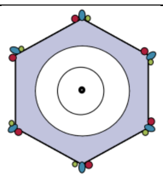
	hepatocytes – Fe^{3+} aggregates	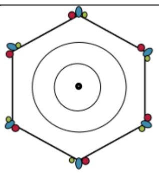	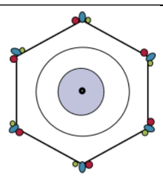	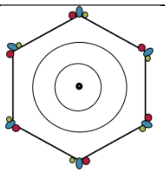	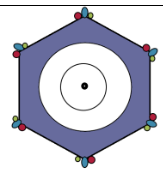
H&E	microvascular steatosis	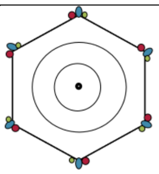	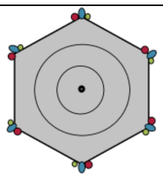	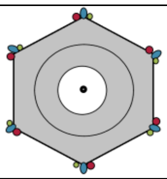	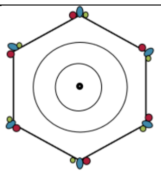

	venous congestion	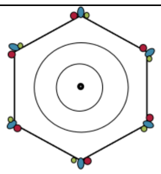	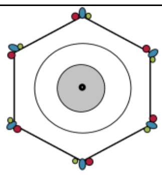	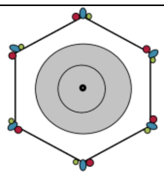	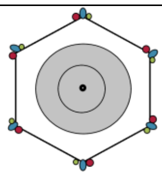
	degeneration of hepatocytes	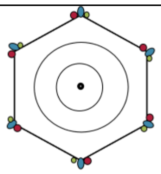	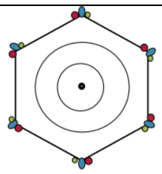	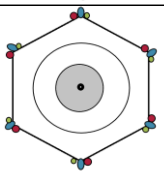	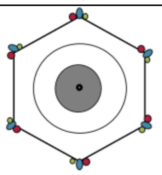
	hemosiderin	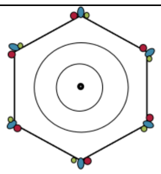	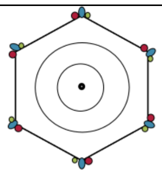	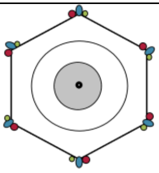	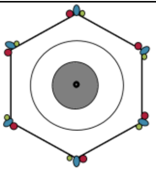

Fe staining: - + ++ +++

H&E staining: - + ++ +++

no lesion (-), mild lesion (+), moderate lesion (++), and severe lesion (+++).

Supplementary Table S5: Immunohistochemical findings (detection of ferritin) in hepatic lobule (liver sections) from sham-operated and HF pigs (mild, moderate, and severe HF groups).

		CONTROL	MILD HF	MODERATE HF	SEVERE HF
FTL	Hepatocytes	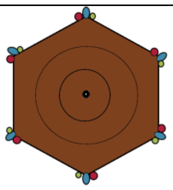	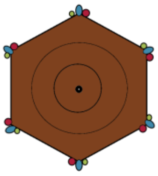	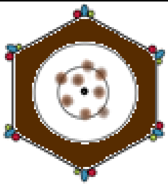	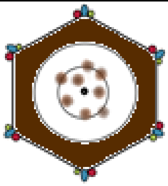
	Kupffer cells	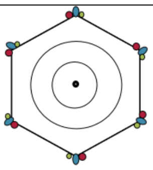	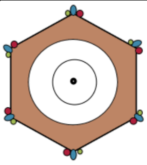	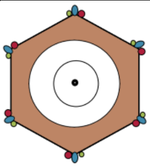	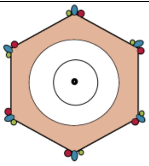
FTH	Hepatocytes	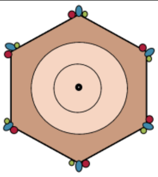	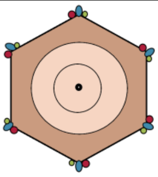	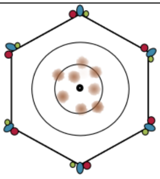	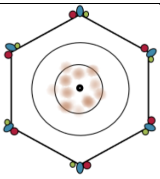
	Kupffer cells	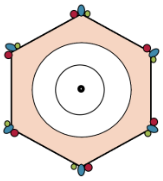	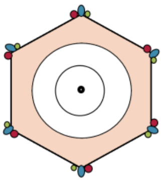	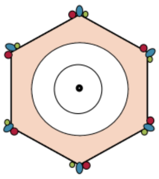	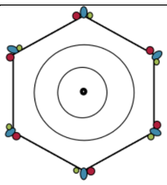

IHC staining: - + ++ +++

no lesion (-), mild lesion (+), moderate lesion (++), and severe lesion (+++).

Supplementary Table S6: Oligonucleotide primers used in RT-PCR experiment

Gene	Sequence 5′-3′	GenBank accession no.
GAPDH	TCACTGCCACCCAGAAGA	AF017079
	TACCAGGAAATGAGCTTGAC	
HAMP	CTCCGTTCTCCCATCCCAGA	AF516143
	TGGGGAAGTGGGTGTCTCTT	

GAPDH – glyceraldehyde-3-phosphate dehydrogenase; HAMP – hepcidin;
